# Feasibility and acceptability of remote administration of the cold pressor test

**DOI:** 10.3389/fpain.2024.1421709

**Published:** 2024-08-20

**Authors:** Jessica K. Salwen-Deremer, Jamie M. Horrigan, Sarah J. Westvold, Jennifer A. Haythornthwaite

**Affiliations:** ^1^ Departments of Psychiatry & Medicine, Dartmouth Hitchcock Medical Center, Lebanon, NH, United States; ^2^ Department of Medicine, Dartmouth-Hitchcock Medical Center, Lebanon, NH, United States; ^3^ Department of Internal Medicine, Yale School of Medicine, New Haven, CT, United States; ^4^ Department of Psychiatry & Behavioral Sciences, Johns Hopkins School of Medicine, Baltimore, MD, United States

**Keywords:** quantitative sensory testing, pain, inflammatory bowel disease, remote monitoring, telehealth, psychogastroenterology

## Abstract

**Objective:**

Quantitative sensory testing is often used to investigate pain in the context of experimental and clinical research studies. However, many of the devices used for QST protocols are only available in resource rich environments, thereby inadvertently limiting the possible pool of participants. Development of remote protocols for appropriate QST measures has the potential to reduce barriers to participation in research.

**Methods:**

Participants with insomnia and Crohn's disease were recruited as part of a clinical trial. We adapted a remote version of the cold pressor test for use during telehealth-based study assessments. Herein, we present data from the baseline assessments including an assessment of feasibility and acceptability of the task.

**Results:**

100% of participants (*N* = 28) were able to complete the remote cold pressor test using a combination of materials from their homes and mailed by the study team. Temperature changes during the test were minimal and fairly evenly balanced between increases and decreases. Correlations between submersion time and both general and disease specific pain trended toward significance.

**Conclusions:**

We demonstrated that a remote version of the cold pressor test is feasible and acceptable in a clinical population and provided a step-by-step protocol for administration to facilitate use in other studies.

## Introduction

Quantitative sensory testing (QST) refers to a group of psychophysical methods to quantitatively and subjectively evaluate individuals' pain experiences. QST approaches may rely on mechanical, thermal, chemical, or electrical stimuli applied under standardized testing protocols to assess local and central pain processing (1, 2). QST is one of the most widely used measures of pain in research studies (1, 3). While all pain assessment is subjective, QST methods enable researchers to compare responses to *standardized* stimuli within and between participants, leading to more objective measures of pain, including changes with treatment, than can be determined based on clinical assessments alone.

Unfortunately, many of the QST protocols use specific devices that require lab personnel and/or expensive equipment for administration (e.g., thermodes for heat-pain tolerance; an algometer for pressure-pain tolerance). Thus, this gold-standard pain assessment is only accessible in resource-rich environments. While other investigators have made efforts to simplify QST protocols and/or use less expensive devices (4), these protocols still require in-person, face-to-face administration. In other areas of investigation, objective remote measurement techniques have been expanded and validated for home use. For example, therapeutic drug levels, obstructive sleep apnea, and blood and stool proteins can all be assessed within patients' or participants' homes using advanced techniques or devices (5–8). However, pain research has yet to advance at a similar rate. Already, participation in research can be difficult for many individuals, particularly patients living in rural regions (9). As a result of the reliance on in-lab QST, experimental and clinical researchers investigating pain sensitivity and its clinical correlates may inadvertently be limiting participant pools and therefore generalizability. It is unlikely and impractical for these protocols to be administered validly outside of a lab setting until new technology is created.

One widely used QST assessment, the cold pressor test (CPT), requires only an ice bath and thus has the potential for use in novel and/or remote settings. While one group has developed a remote CPT administration protocol (10), their participants were healthy controls and did not endorse regular pain. Additionally, in consultation with the lead author of that group, he expressed some concern about ice melting and water temperature rising. Finally, as that protocol was fully asynchronous and completed without precise measures of temperature, the authors were unable to report on stability of the testing. Thus, we sought to refine their protocol and test its feasibility in a clinical population.

## Method

### Participants

Participants were recruited from the Dartmouth-Hitchcock Medical Center Inflammatory Bowel Disease Center as part of a parent clinical trial on Cognitive Behavioral Therapy for Insomnia (CBT-I) in people with Crohn's disease (NCT05034159). Data presented herein were collected as part of this trial's baseline assessment, and represent an embedded feasibility and acceptability assessment of a study task. This study was approved by the Dartmouth Health Human Research Protection Program (#02001191) and informed consent was provided by all participants. Participants provided informed consent, including verbalized understanding of the study protocol during a phone call followed by a signature and acceptance of an electronic consent form.

Efforts were made to reduce barriers to participation in order to widen our potential participant pool, including minimizing the number of in-person visits (2–3 visits over 4–7 months), conducting study visits outside of normal business hours, and pairing study visits with other required medical visits to reduce travel, among other strategies. Recruitment occurred between October 22, 2021 and September 18, 2022 and inclusion criteria were as follows: (1) mild to moderate Crohn's disease as assessed by the Patient Reported Outcomes-3 (11), (2) Insomnia Severity Index (12) score ≥8, (3) sleep onset latency and/or wake after sleep onset ≥30 min, (4) willingness to not change sleep medications over the course of the trial, and (5) access to a device and internet or cell phone service sufficient for telehealth visits. Exclusion criteria were as follows: (1) PHQ-9 (13) depression score ≥20, (2) GAD-7 (14) anxiety score ≥20, (3) unstable major psychiatric condition, (4) current alcohol or substance abuse, (5) current opioid use for pain control, (6) current smoker (tobacco, nicotine), (7) current systemic corticosteroid use, (8) current pregnancy or nursing, (9) ileostomy or colostomy, (10) diagnosis of seizure disorder, (11) diagnosis of sleep apnea or positive STOP-Bang (15) screen, (12) diagnosis of restless leg syndrome or positive Cambridge-Hopkins RSLq (16) screen, and (13) night shift, rotating shift work, or frequent travel outside of primary time zone. Recruitment stopped after we had reached our planned sample size of 26 randomized participants. This target was determined based on anticipated effect sizes for the primary outcomes (changes in sleep continuity) in the parent clinical trial.

### Outcomes

The feasibility of the remote CPT was determined based on (1) whether requisite supplies were attainable from participants' homes, (2) set-up in participants' homes was convenient, (3) instructions could be executed remotely with good compliance, (4) ice remained in 100% of participants' containers at 2 min, and (5) no participants expressed distress or other adverse events. The acceptability of the remote CPT was determined based on (1) a minimum of 80% of participants completing the task without significant distress or concern, (2) at least 80% of participants would be willing to repeat the task during the subsequent assessment period, and (3) a 2-minute task duration limit would maintain variability in responses, while reducing the likelihood of distress.

### Measures

After participants consented and screened into the study, they completed a comprehensive baseline assessment including self-report questionnaires, objective sleep continuity and sleep architecture assessments, blood and stool testing, and the remote cold pressor test. Study boxes containing all necessary research devices were mailed to participants, and device use and stool collection guidelines were reviewed during a synchronous audio and video telehealth visit. Administration of the cold pressor test also took place during this telehealth visit. Measures included herein were as follows:

#### Demographics

Participants self-reported their age, gender, race, and ethnicity. Distance to the medical center was calculated based on their home address.

#### Social deprivation index (SDI)

This index was developed by the Robert Graham Center and serves as a measure of social determinants of health (17). Scores are calculated based on 2019 American Community Survey zip code level data and greater scores are indicative of worse overall social deprivation. SDI scores have been shown to correlate with poor access to care and poor health outcomes (18).

#### Brief pain inventory

This questionnaire was used to assess bodily pain in the last week (19). Participants rated their worst, least, average, and current pain on a scale from 0 (no pain) to 10 (worst pain imaginable). Ratings across these 4 areas were averaged to form an overall pain intensity score. Participants also rate the degree to which pain interferes with 10 aspects of daily life. Ratings across these areas were averaged to form an overall pain interference score.

#### Patient-Reported outcomes-3

In this 3-item questionnaire, participants report their number of liquid or soft stools per day, the severity of their abdominal pain, and their overall well-being averaged over the last week (11). Each item has a different scoring system, and item scores are combined for an overall assessment of disease activity.

### Remote cold pressor test administration

Notably, we made several changes to McIntyre et al.'s protocol, including: (1) measuring temperature before and after the task with a standardized thermometer so we could use temperature change as a covariate, (2) requiring water temperature be <40 °F (4.4 °C) to ensure the water was cold enough to produce discomfort, (3) doubling the amount of ice used to ensure it did not melt during the task (by filling ¼ of the container with ice instead of ⅛), and (4) changing from 2.5 to 2 min of maximum submersion time. In addition, while McIntyre et al. asked participants to report on their “maximum pain intensity”, we chose to use the language “as painful or as uncomfortable as you can imagine” to describe the anchor point of 10 on a 0–10 scale. We made this change in language in order to (1) better capture the variety of language that people with chronic, painful conditions use to describe their pain experiences (20–24), and (2) create a description that was more culturally inclusive (25).

#### Preparation

Included in their study device box was a submersible digital thermometer that was accurate within 1 ° Fahrenheit. They were also asked if they had a way to make at least 2 “trays worth” of ice cubes (e.g., two 4.75″ × 12.5″ × 1.5″ trays, each of which produce 16 ice cubes). If they did *not* have access to ice, the study team included 3 ice cube trays in the mailed box.

#### Administration

1. Participants were instructed to find a large cooking pot, mixing bowl, bucket, or similar container and a towel. They were told the bowl should be big enough that they could fully submerge their hand inside without touching the sides of the bowl. The coordinator verified the bowl was appropriately sized before proceeding to the next step.
2. Participants were instructed to prepare the cold water bath as follows:
  a. Fill the bowl about a quarter of the way full with ice.
  b. Turn on the sink water, turn it to cold, testing with fingers from either hand.
  c. Fill the bowl with cold water until it is about three quarters of the way full.
  d. Stir the water with a large spoon.
  e. Insert the thermometer into the water (not touching the bottom or sides of the bowl) and tell the coordinator what it reads.
3. If after water bath set up the water temperature was ≥40 °F (4.4 °C), participants were instructed to add more ice and repeat steps d and e above.
4. Participants were then read the following instructions: *Thank you for getting everything ready. In a moment, I'm going to tell you to put your hand in the water—I will say go when it is time. It should be your non-dominant hand, so, not the one you write with. I will start my timer as soon as your hand goes in the water. Periodically, I’m going to ask you to rate your discomfort from 0, where your hand feels absolutely fine, to 10, where it feels as painful or as uncomfortable as you can imagine. I'll prompt you each time by saying the word “rating”. Keep your hand in the water as long as you can tolerate, even if it gets uncomfortable. If you reach the two-minute mark, I'll ask you to remove your hand at that point. Once you take it out, you are all done and you can dry it on the towel. Again, try to keep your hand in for as long as you can! Do you have any questions before we begin?*
5. Once the participant was ready, the coordinator asked them to go ahead and requested ratings every 20 s. They recorded ratings (0–10) at each 20 s interval, the time at which participants removed their hands, and a final discomfort rating.
6. After participants removed their hands, they were asked to take a final temperature reading and to tell the coordinator whether they had ice left in their container.
7. Two final pain-specific variables were computed, defined as follows:
  a. ***Maximum discomfort:*** the highest discomfort rating (from 0 to 10) reported by a participant during the time their hand was submerged.
  b. ***Submersion time:*** the total time the participant kept their hand submerged in the cold-water bath, with a maximum time of 2 min.
8. Change in water temperature both in degrees and as percent change were also computed.

#### Data analysis

All CPT data were entered into an excel spreadsheet and reviewed for completeness and accuracy by the research coordinator during the time of testing. Self-report questionnaires were reviewed for missingness, skew, and kurtosis. There were no missing data and all data were normally distributed with the exception of distance from the medical center. CPT variables (start temperature, end temperature, submersion time, and maximum discomfort) were also normally distributed. Descriptive (means, standard deviations, and percentages) and inferential statistics (Pearson correlations) were completed with IBM SPSS version 29.

## Results

Participants included 28 people with Crohn's disease and insomnia with ages ranging from 20 to 72 (M = 42.79, SD = 13.78). Participants with Raynaud's or other similar vascular conditions that could be significantly worsened or exacerbated by participation in this task were exempt. With regard to cardiac conditions, one participant endorsed current postural orthostatic tachycardia syndrome and another endorsed unspecified tachycardia. Both of these diagnoses were also listed in their medical records. All participants elected to participate in the CPT task. Participants were predominantly women (64.3%) and non-Hispanic white (89.3%). Participants were recruited from 3 states and from a range of 4.1 to 221 miles from the medical center (M = 62.57, SD = 43.07). Social deprivation index scores ranged from 2 to 85 (M = 32.93, SD = 25.32), with 4 participants (14.3%) scoring in the high social deprivation range, 13 (46.4%) in the intermediate range, and 11 (39.3%) in the low range. Demographic information is presented in Table 1.

**Table 1:** Basic demographics and descriptive statistics for the remote cold pressor test (*N* = 28).

Participant demographics	
Age	42.79 years (13.78)
Gender	64.3% women
Race	89.3% white
Distance from medical center	62.57 miles (43.07)
Social deprivation index	32.93 (25.32)
Crohn's disease severity (PRO-3)	22.86 (11.51)
Pain intensity (BPI)	3.89 (2.29)
Pain interference (BPI)	4.13 (3.21)
Cold pressor test (CPT) characteristics	
Start temperature	36.02 °F (3.01)
End temperature	36.06 °F (2.55)
Maximum discomfort	8.14 (1.88)
Submersion time	101.89 s (34.03)

Based on our pre-determined criteria, feasibility and acceptability of the remote CPT were reached. Overall, 100% of participants were able to find an appropriately sized bowl, gather enough ice to fill a quarter of their chosen bowl, and complete the task as instructed. 100% of participants had ice remaining at the end of the task. Mean water temperature was 36.02 °F (SD = 3.01) at the beginning of the task and 36.06 °F (SD = 2.55) at the end of the task (Table 1). The correlation between water temperature at the beginning and end of the task was.66 (*p *< .001) and the temperature change from beginning to end ranged from 0 °F to 5.9 °F (absolute value of change mean = 4.8%, median = 3.2%, SD = 4.11). Temperature decreased over the two minutes for 15 participants (53.6%), stayed the same for 1 participant, and increased for 12 participants (42.9%). 85.7% of participants had a change of ≤3 °F in temperature. Neither starting temperature nor temperature change (in degrees F) were significantly related to submersion time (*p *= .73 and *p *= .65, respectively) or to maximum discomfort (*p *= .23 and *p *= .49, respectively).

Participants reported a mean maximum discomfort of 8.14/10 (SD = 1.88) and maximum discomfort scores ranged from 3 (1 participant) to 10 (7 participants). 78.5% of participants rated their overall maximum discomfort as an 8/10 or greater. 21/28 (75%) participants kept their hand submerged for the full duration (2 min), with the remaining 7 participants' submersion times ranging from 14 s to 1 min and 26 s. Overall mean submersion time was 101.89 s (SD = 34.03).

Correlations between submersion time and measures of both general pain (BPI pain intensity subscale) and disease-specific symptom severity (PRO-3) trended toward significance (*r *= −.35, *p *= .065; *r *= −.33, *p *= .08, respectively). However, maximum discomfort was not significantly correlated with either (*r *= .26, *p *= .19 and *r *= .23, *p *= .25, respectively). Scatter plots of these relationships are included in the Supplementary Figures S1–S4) to allow for additional presentation of the data. Plots include both a linear regression line and a nonparametric loess line to improve visualization of data trends, though given our small sample and the restricted range in submersion time, further investigation of the line of best fit for these relationships is warranted. Neither submersion time nor maximum discomfort were associated with pain interference (BPI; *p*'s > .36).

A subset of participants (*n* = 12) were invited to complete the CPT 12 weeks later, following a waitlist control period. Procedures for this follow-up CPT were identical to the first. The two maximum discomfort scores were significantly correlated (*r *= .64, *p *= .026), as were the submersion times (*r *= .63, *p *= .027). Similarly, intraclass correlation coefficients suggested moderate test-retest reliability for both maximum discomfort (ICC = .63) and submersion time (ICC = .64) (26). Looked at another way, 9/12 participants (67%) had maximum discomfort ratings within 1 point across the two time points and 10/12 participants (83%) had complete agreement between the two submersion time points (2 min submersion for all of these participants).

## Discussion

Herein, we sought to establish the feasibility and acceptability of remote completion of the cold pressor test in a clinical population—clinical trial participants with symptoms of insomnia and physician diagnosed mild- to moderately-severe Crohn's disease. McIntyre and colleagues demonstrated that the remote cold pressor test performs similarly to the in-lab cold pressor test (10), and our research expands their work, demonstrating that the test is also feasible and acceptable in a clinical population.

All participants had the requisite supplies in their home (large container, ice), were able to set up and complete the task as instructed, and had ice remaining in the container at the end of the task. In a subset of participants, we also found the remote cold pressor test to demonstrate moderate test-retest reliability. Reliability ratings herein were consistent with lab-based measures of both the CPT and quantitative sensory testing more broadly (27, 28). Finally, we found that submersion time trended toward significance in investigations of correlations with both disease-specific symptom severity and general measures of pain. While maximum discomfort was not significantly correlated with self-report measures of pain, the correlations themselves were small to moderate and in the expected direction, and may have been non-significant due to our small sample.

Notably, we made several changes to McIntyre et al.'s protocol. As described above, while McIntyre et al. used a 2.5-minute maximum submersion time, we used 2 min in an attempt to reduce participant distress in the context of a painful chronic disease. However, as no one expressed distress and 77.8% of participants kept their hand submerged for the entire two minutes, we plan to extend the submersion window for submersion to 3 min in future trials to balance addressing this ceiling effect with participant burden and discomfort.

Conducting study assessments remotely using protocols such as the remote CPT protocol described herein has the potential to reduce transportation barriers for participants, thereby widening the possible participant pool and improving generalizability of findings. Risk for chronic pain is increased in people with lower socioeconomic statuses and people from marginalized or minority groups (29–31), some of which may be explained by limited access to healthcare, including access to specialists (32). Recently, there have been calls to confront racism in pain research (33), including recommendations to reduce barriers to trial involvement (34). The mitigation of barriers to participation through improvement of remotely administered assessments has the potential to improve health outcomes in all patients, particularly the most vulnerable.

Despite the strengths and value of this work, it is not without limitations. While our participants were drawn from a large area around the medical center and distributed widely across the social deprivation index, they were ethnically/racially homogeneous, highlighting the need for further investigation of this adapted protocol in a larger, more diverse sample. Further, while we were unable to rely on water circulation to maintain a stable temperature during the test, temperature changes were minimal and fairly evenly balanced between increases and decreases. Unfortunately, research suggests that across the literature, it is common for articles to not report whether a specific temperature range was maintained or if the water was circulated (35),—thus it is unclear how common water temperature variation is across the literature. We were also unable to standardize the testing environment, although prior research has also demonstrated that room temperature and humidity do not significantly impact CPT stability (36), further supporting the use of in-home testing. In addition, nonstandardized cold water vessels may have led to differences in heat transfer; while one could consider mailing participants vessels or requiring use of a specific material, this degree of standardization would increase cost and burden, likely with minimal improvement in rigor. Recent research indicates that reported pain sensitivity during the CPT is not significantly related to skin temperature changes (37), further supporting the choice to use nonstandardized vessels over the possible gains from standardization. Finally, we did not investigate the ability of the remote CPT to predict changes over time. Other QST protocols, including the cold pressor test, have been shown to predict longitudinal outcomes, such as pain at follow-up (2). Similarly, single-session intervention studies using various psychological techniques (e.g., hypnosis, virtual reality) have demonstrated improvements in pain tolerance, as measured by the CPT (38, 39). Future research investigating not only whether a remote cold pressor test is responsive to intervention-related changes in pain, while also providing information distinct from patient reported outcomes, will be critical for understanding the clinical utility of this task.

## Conclusions

Overall, we demonstrated that a remote version of the cold pressor test is feasible and acceptable in a clinical population. Submersion time also correlated with self-report measures of general and disease-specific pain. Use of in-home measures of pain tolerance in clinical trials in lieu of lab-based measures has the potential to reduce barriers to participation, supporting initiatives to improve access to and participation in clinical trials in marginalized and/or minority populations.

## Data Availability

The datasets presented in this article are not readily available because the data contain sensitive information that could compromise the privacy of research participants. Requests to access the datasets should be directed to jessica.k.salwen-deremer@hitchcock.org.
