# Supplementary material for: Feasibility and acceptability of remote administration of the cold pressor test
Source: Front Pain Res (Lausanne). 2024 Aug 20;5:1421709. doi: 10.3389/fpain.2024.1421709 (PMC11368846; doi:10.3389/fpain.2024.1421709)

Supplementary Figure 1. Scatter plot of the relationship between Cold Pressor Test (CPT) submersion time and pain severity, as measured by the Brief Pain Inventory (BPI).

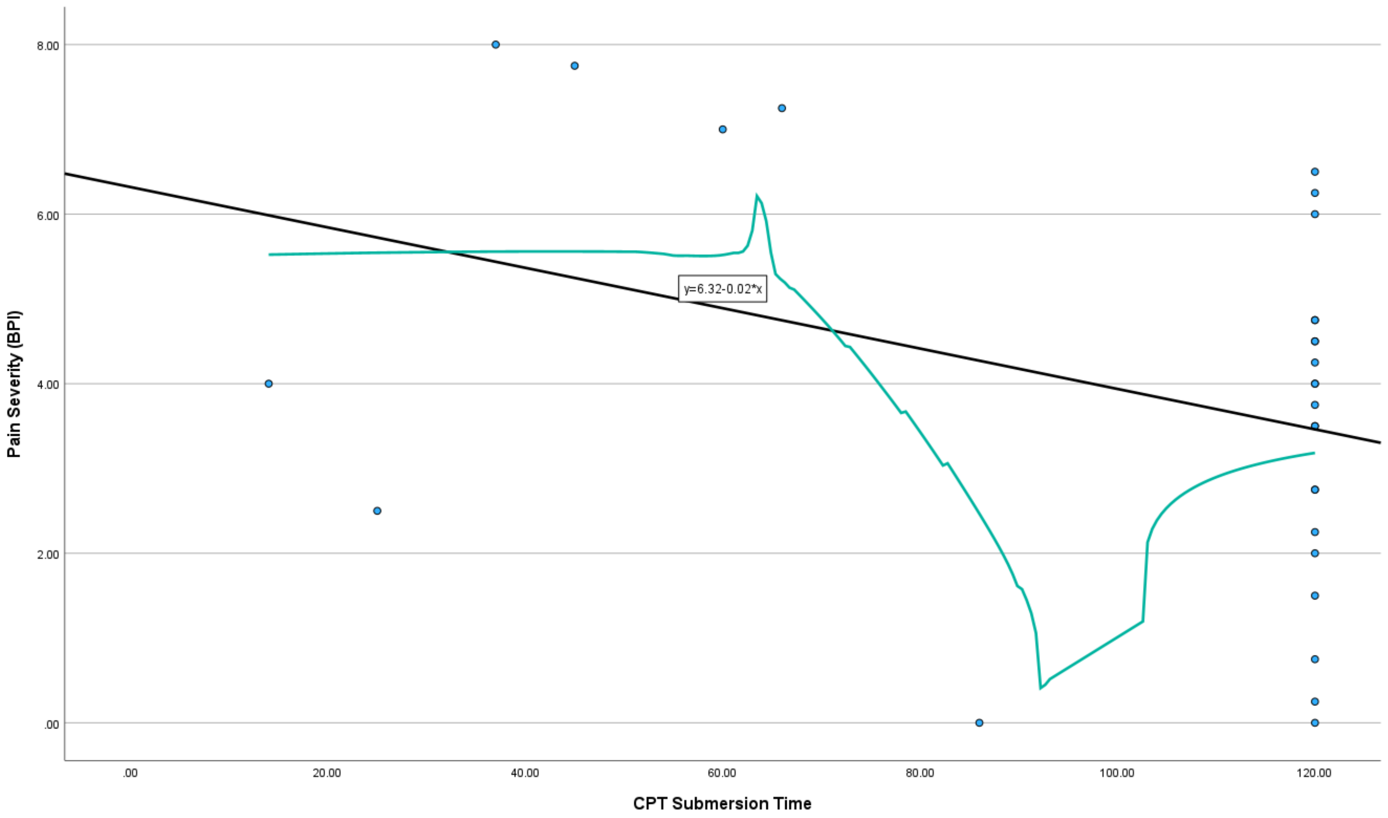

Supplementary Figure 2. Scatter plot of the relationship between Cold Pressor Test (CPT) submersion time and Crohn's disease symptom severity, as measured by the Patient Reported Outcomes 3 (PRO-3).

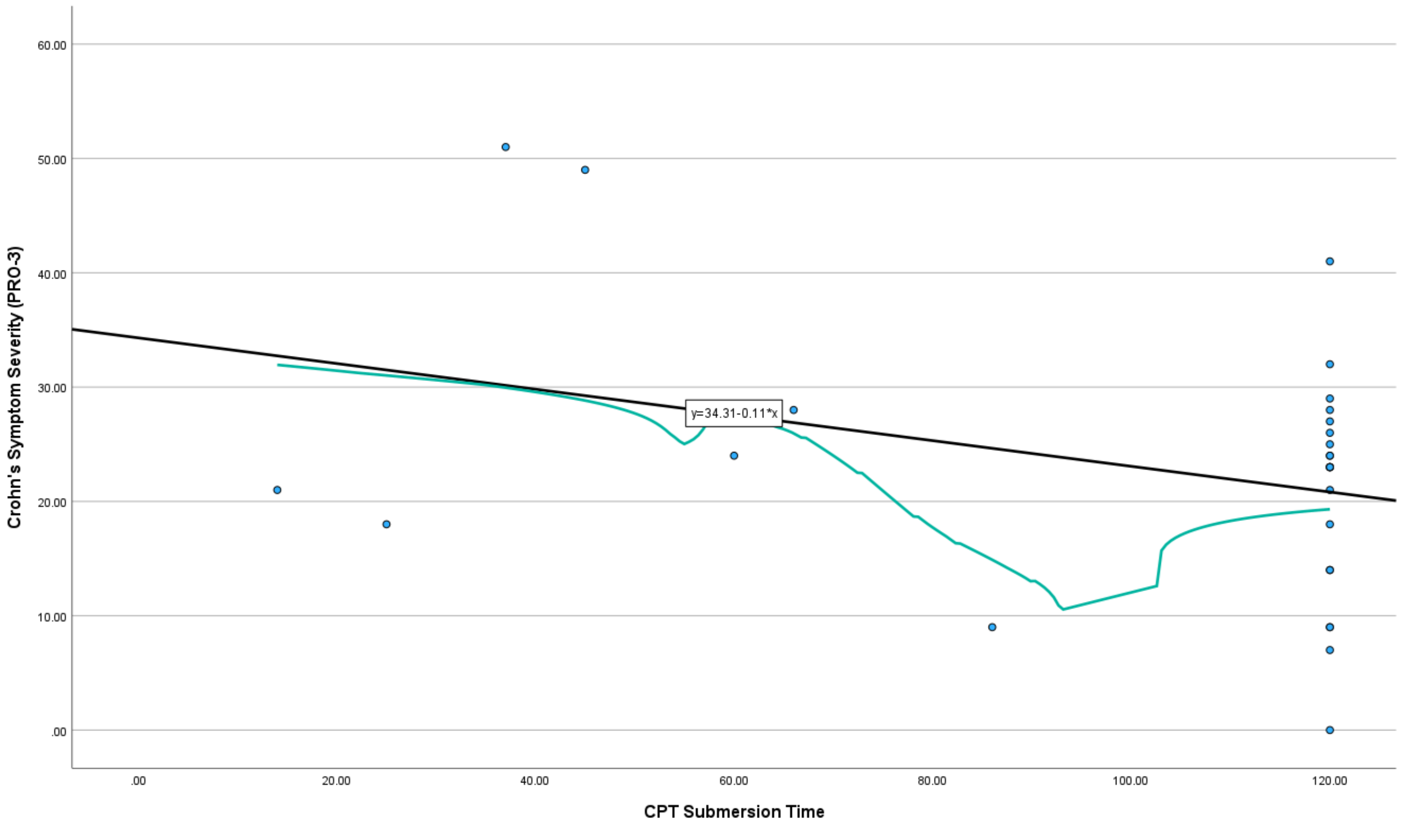

Supplementary Figure 3. Scatter plot of the relationship between Cold Pressor Test (CPT) maximum discomfort rating and pain severity, as measured by the Brief Pain Inventory (BPI).

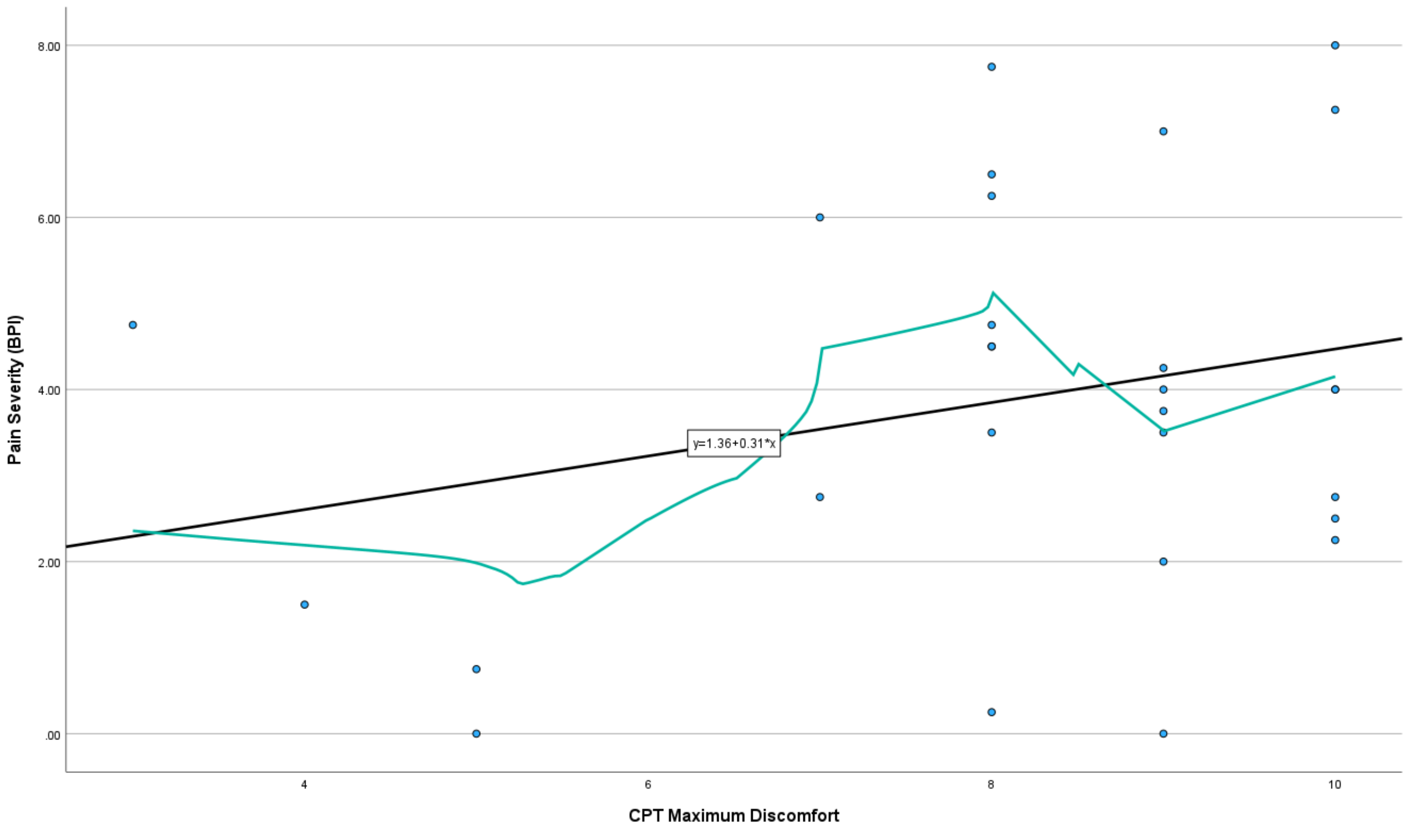

Supplementary Figure 4. Scatter plot of the relationship between Cold Pressor Test (CPT) maximum discomfort rating and Crohn's disease symptom severity, as measured by the Patient Reported Outcomes 3 (PRO-3).

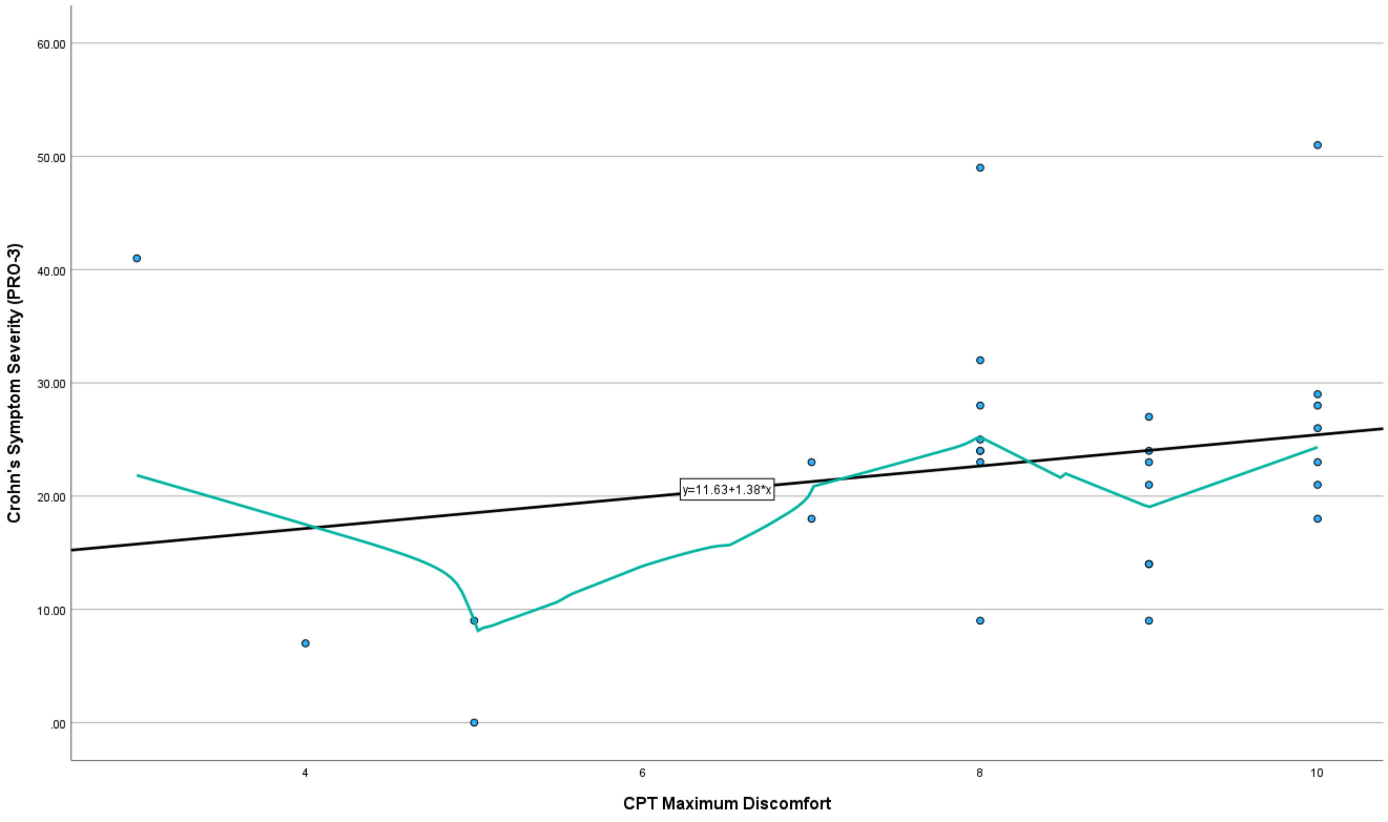

Supplement: Supplementary file 1 [file Datasheet1.pdf]
